# On the Best Method of Detecting Small Quantities of Albumen in the Urine

**Published:** 1866-05

**Authors:** Andrew Clark

**Affiliations:** Assistant Physician to the London Hospital


### Miscellaneous.

### On the best Method of Detecting small quantities of Albumen in the Urine.

By Dr. Andrew Clark, Assistant Physician to the London Hospital.

It is of great clinical importance to possess a simple and certain method of detecting small quantities of albumen in the urine. After a great variety of experiments, I have come to the conclusion that nitric acid used in the manner about to be described—a modification of the plan suggested by Heller—is by far the most sensitive, reliable and handy agent that can be used for this purpose by the physician.

Pour not less than half a drachm of *fuming* nitric acid into a test tube; incline it, and then let a like quantity of the suspected urine trickle down *very slowly* to the acid, over the surface of which the urine will float without the slightest admixture. If albumen be present, a milk-white, sharply-defined, tolerably tenacious film will form at the exact point of junction of the two fluids. This film is never, at first, thick; and when the amount of albumen in the urine is extremely minute, it may be so thin as to become visible only by reflected light when the test tube is inclined. Occasionally, when very thin, the albuminons film is dissolved in the course of a few hours. Commonly, however, it increases in breadth, diminishes in density, becomes yellow or yellowish-green at its under surface; and throws off minute coagula, which fall through the acid to the bottom of the tube.

Nitric acid used in this manner as a test for albumen is also a test of the presence of uroxanthine, or bile—either or both of which are not unfrequently present in temporary and functional albuminuria.

If, in immediate contact with the acid, a ruby or violet ring is developed, uroxanthine is present; and bile also, if in addition to a red or violet there is formed likewise a green-colored ring, which remains for some time.

Two feasible objections are urged against depending solely on the employment of nitric acid in the manner described, as a test of the presence of albumen; and I have myself noticed a third; but a careful examination of their force leads me to a conclusion that they are more theoretical than real.

When urine, rich in uric acid or its salts, or containing much scaly epithelium, is poured over cold nitric acid, a general turbidity arises, which is said to be undistinguishable from that produced by the presence of albumen.

But if the proposed test for albumen be properly applied, no turbidity will be produced by the presence of that substance, unless urates are also present. And then the white film of albumen is separated from the superimposed turbidity by a thin stratum of clear urine.

The turbidity produced by uric acid or epithelium is general, granular-like, and without any approach to coherence.

The turbidity approached by urates is sometimes abruptly defined below by an opaque, ring-like border, sometimes colored, sometimes not; but a stratum of clear urine intervenes between this ring and the surface of the acid, and, as above, the turbidity has no cohesion of parts. Besides this, the turbidity produced by lithates may be immediately dissipated by heat; and, if not in great excess, even by the heat of the hand closed around the tube.

The film produced by the contact of nitric acid with albuminous urine is quite different from any kind of turbidity. Confined to the layer of urine resting upon the acid, white like the disc of compressed cotton, tenacious, and when shaken with its associated fluids, breaking into flaky fragments, it seems improbable that any but the merest tyro should mistake it for anything but what it is.

In testing for albumen by means of heat and nitric acid, there may be no immediate response indicative of its presence; and yet after a few hours a flocculent precipitate may form and fall to the bottom of the tube.

A specimen of urine examined within an hour after extrusion from the bladder may yield unequivocal evidence of the presence of albumen, and cease to do so after twelve hours.

Small films of coagulated albumen produced on the surface of nitric acid occasionally disappear within a few hours from the time of their formation.

Little importance is to be attached to the presence of small quantities of albumen in the urine of women a day or two before or after menstruation. It is common without any disorder of the kidney or any sensible discharge from the vulva.

Small quantities of albumen are often present in the urines of women with leucorrhœa, and of those who have recently had fits of hysteria.

One is not justified in asserting the absence of albumen in the urine upon the result of one or two examinations. I knew a case in which albumen occurred in the urine daily for several months; but it was present only in the urine first passed after breakfast, and was never, to the time of its departure, present in the urine passed at any other time.

Men sometimes discharge a thin, whitish, glairy fluid, with the closing stream of urine in the act of emptying the bladder. This fluid is said to be seminal; but in none of the examples that I have examined were any spermatic filaments present. From its containing mucin, and young cell particles, I look upon it as an augmented and slightly altered secretion of the glands opening into the urethra. When discharged in any quantity, the urine containing it responds to all the ordinary tests of the presence of albumen.

Albumen in small quantities and unoccupied by casts, may be present in the urine daily for three years, and at last permanently disappear. This occurred in a case under my observation. The health which had previously been bad, rapidly improved after the disappearance of albumen from the urine, and became ultimately very good.

Mere hepatic congestions is sometimes the cause of slight functional albuminuria. I had under observation for sometime a lady whose "liver attacks" were invariably preceded by the appearance of small quantities of albumen in the urine. With the free purgation which was found necessary for the removal of these attacks, the albumen disappeared. I remember also the case of a gentleman who was subject to somewhat similar attacks. In his urine, however, free uric acid was associated with the albumen, and both stayed several days beyond the subsidence of acute disorder. But he was a wilful patient, and chose to live well even at the cost of being ill.—*Clinical Lectures and Reports of London Hospital*, 1864, *p.* 224.

